# Beyond BCMA: the next wave of CAR T cell therapy in multiple myeloma

**DOI:** 10.3389/fonc.2024.1398902

**Published:** 2024-05-10

**Authors:** Kevin Miller, Hamza Hashmi, Sridevi Rajeeve

**Affiliations:** Myeloma Service, Department of Medicine, Memorial Sloan Kettering Cancer Center, New York, NY, United States

**Keywords:** multiple myeloma, CAR T cell therapy, chimeric antigen receptor, non-BCMA, immunotherapy

## Abstract

Chimeric antigen receptor (CAR) T cell therapy has transformed the treatment landscape of relapsed/refractory multiple myeloma. The current Food and Drug Administration approved CAR T cell therapies idecabtagene vicleucel and ciltacabtagene autoleucel both target B cell maturation antigen (BCMA), which is expressed on the surface of malignant plasma cells. Despite deep initial responses in most patients, relapse after anti-BCMA CAR T cell therapy is common. Investigations of acquired resistance to anti-BCMA CAR T cell therapy are underway. Meanwhile, other viable antigenic targets are being pursued, including G protein-coupled receptor class C group 5 member D (GPRC5D), signaling lymphocytic activation molecule family member 7 (SLAMF7), and CD38, among others. CAR T cells targeting these antigens, alone or in combination with anti-BCMA approaches, appear to be highly promising as they move from preclinical studies to early phase clinical trials. This review summarizes the current data with novel CAR T cell targets beyond BCMA that have the potential to enter the treatment landscape in the near future.

## Introduction

The treatment landscape of multiple myeloma has vastly changed over the past two decades with the introduction of novel classes of drugs which significantly improved survival outcomes. More recently, the emergence of immunotherapies including chimeric antigen receptor (CAR) T cell therapy further expanded the myeloma treatment armamentarium (1–7). CAR T cells are cellular therapy products derived from the *ex vivo* genetic modification of T cells with a CAR construct. CARs are modular transgenes comprised of a target-binding domain that recognizes cell surface molecules in a major histocompatibility complex-independent fashion, a hinge or spacer domain, a transmembrane domain, co-stimulatory domain(s) such as CD28 or 4-1BB, and a CD3ζ signaling domain (8). After *ex vivo* expansion, CAR T cells are infused into lymphodepleted patients. Upon target engagement, CARs activate T cells, causing them to destroy tumor cells bearing their cognate antigen. To date, CAR T cell therapy has been clinically effective in several hematologic malignancies leading to durable responses in a subset of treatment refractory patients (9). However, apart from logistical and financial challenges associated with creating and administering these autologous therapeutics, other limitations include unique toxicities such as cytokine release syndrome (CRS) and immune effector cell associated neurotoxicity syndrome (ICANS), which can cause significant morbidity and in some cases be fatal (10). In addition, there are significant risks of infection and persistent cytopenias after CAR T cell therapy (11–13). Thus, administering CAR T cell therapy is a cost- and labor-intensive endeavor that requires significant multi-disciplinary expertise (14). Despite these limitations, the promise of living drugs that can expand *in vivo*, eliminate tumor cells, and potentially persist for years – the fruits of decades of research – has generated immense enthusiasm.

There are currently two Food and Drug Administration (FDA) approved CAR T cell therapies for the treatment of relapsed/refractory (R/R) myeloma: Idecabtagene vicleucel (ide-cel) and ciltacabtagene autoleucel (cilta-cel), which were first approved in March 2021 and February 2022, respectively, for use in patients treated with at least 4 prior lines of therapy (4, 15). Both ide-cel and cilta-cel target B cell maturation antigen (BCMA), which is a tumor necrosis factor superfamily receptor expressed almost exclusively on human plasma cells that has a functional role in myeloma tumorigenesis (16–18). Ide-cel was the first FDA approved CAR T cell in myeloma and bears a murine single chain variable fragment (scFv) anti-BCMA target-binding domain as well as a 4-1BB co-stimulatory domain. Initial FDA approval was based on the phase 2 KarMMa study reported by Munshi et al., where in 128 infused patients with R/R myeloma, ide-cel demonstrated an overall response rate (ORR) of 73%, with a complete response (CR) rate of 33%, measurable residual disease (MRD)-negative rate of 26% (from here, defined as less than 1 in 10^−5^ nucleated cells), and median progression-free survival (PFS) of 8.8 months (15). The second FDA approved CAR T cell therapy is cilta-cel, which has a target-binding domain comprised of two camelid heavy-chain only anti-BCMA fragments and includes a 4-1BB co-stimulatory domain. Initial approval was supported by the phase 1/2 CARTITUDE-1 trial, where in 97 infused patients, the response rate was 98% with a CR rate of 83%, MRD-negative rate of 92% (among evaluable patients) and remarkable median PFS of 34.9 months (4, 19, 20).

In April 2024, the FDA revised the label of both ide-cel and cilta-cel to include patients treated with 1-2 prior lines of therapy based on results of two randomized phase 3 trials: KarMMa-3 and CARTITUDE-4, respectively. KarMMa-3 compared ide-cel with standard regimens in patients with R/R myeloma, and demonstrated significant improvements in response rate (71% vs. 42%), MRD-negative rate (20% vs. 1%) and PFS (1-year PFS, 55% vs. 30%) (6). CARTITUDE-4 compared cilta-cel to standard regimens in R/R myeloma, demonstrating an improved response rate (84.6% vs. 67.3%), MRD-negative rate (60.6% vs. 15.6%) and PFS (1-year PFS, 75.9% vs. 48.6%) (7). While the trial designs, patient populations, and prior therapies/refractoriness were slightly different, both showed that anti-BCMA CAR T-cell therapy improved depth and duration of remission compared to standard salvage therapies. These trials established anti-BCMA CAR T cell therapy as superior for patients with daratumumab-refractory disease. However, it remains unclear whether anti-BCMA CAR T cells are better than existing salvage regimens for patients who are daratumumab-naïve or daratumumab-exposed (but not refractory), especially for those with standard-risk disease biology. This question can be addressed by in-depth analysis of the CARTITUDE-4 trial as well as from real-world evidence on the use of anti-BCMA CAR T cells in earlier lines of therapy. Taken together, clearly ide-cel and cilta-cel have revolutionized the treatment paradigm for patients with R/R myeloma. There are also several other anti-BCMA CAR T cell products currently in various stages of clinical development, including several allogeneic products, as well as combination trials such as with an oral γ-secretase inhibitor to increase BCMA surface antigen density (21–36).

Despite remarkable efficacy in R/R myeloma, it is increasingly evident that most patients with deep responses after anti-BCMA CAR T cell therapy subsequently relapse (15, 19). Notably, in the phase 2 trial with ide-cel, almost all evaluable patients (96%) retained BCMA expression by immunohistochemical (IHC) analysis at the time of relapse, suggesting complete loss of BCMA is rare (15). However, there is emerging data that even if BCMA expression is maintained, decreased antigen density may contribute to resistance (37). Interestingly, in the infrequent cases of complete loss of BCMA, acquired biallelic *BCMA* deletions and/or truncating mutations have been described (38–40). In a slightly different context – i.e. relapse after exposure to anti-BCMA bispecific T cell engagers (TCE) – Lee et al. described several patients who developed non-truncating mutations in the *BCMA* extracellular domain that functionally abrogated drug binding despite retained surface protein expression (40). Whether this mechanism contributes to relapses solely after CAR T cell therapy requires further investigation, but was not identified in the aforementioned study. Taking target loss out of the equation, several other factors could contribute to both primary resistance and relapse after anti-BCMA CAR T cell therapy. In fact, the growing experience from patients treated with anti-CD19 CAR T cell therapy for B cell lymphomas has identified the importance of CAR T cell immunophenotypic characteristics, including propensity toward immune exhaustion and complex signaling cross-talk within the tumor microenvironment as major determinants of therapeutic response (41–50). The contribution of these mechanisms toward resistance to anti-BCMA CAR T cell therapy is an area of active investigation (51–55). For example, using single-cell techniques, Freeman et al. recently presented that durable anti-BCMA CAR T cell therapy responders had a lower baseline CD4+ and CD8+ T cell exhaustion signature relative to non-responders (53). Another group presented data showing that anti-BCMA CAR T cells with a predominantly terminally differentiated phenotype and exhaustion signature were associated with poor response, compared with central or effector memory CAR T cells, which were associated with durable response (51). Finally, Ledergor et al. recently showed that patients whose CAR T cells had a CD8+ effector memory cell phenotype had more frequent durable responses, whereas increased exhausted CD4+ CAR T cells were associated with early relapse (55). As further studies are published in this in the coming years, the hope is that immunologic features associated with poor response may be further clarified and potentially mitigated with novel techniques.

**Figure 1:**
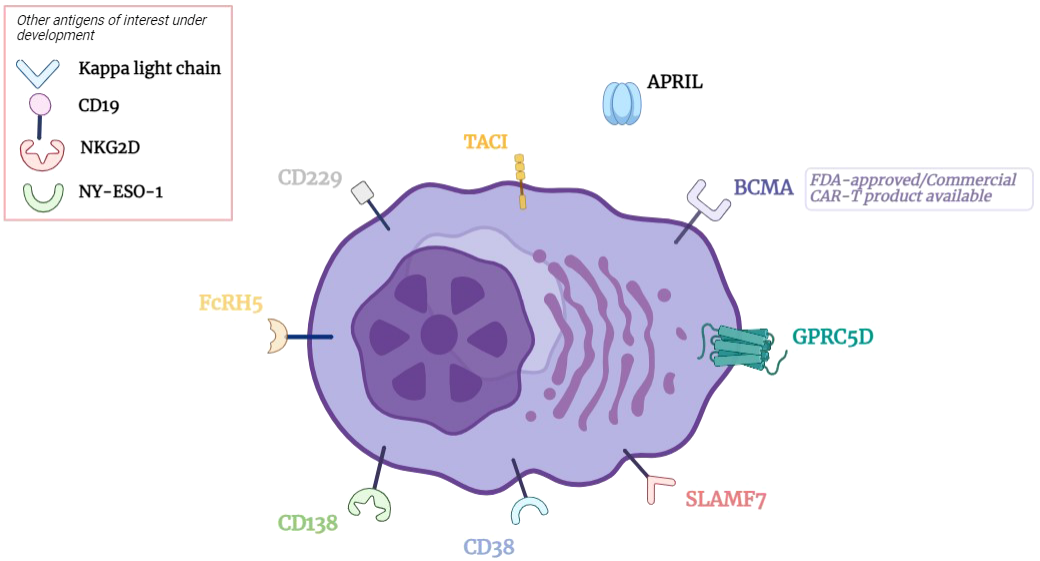
CAR T cell Therapy Targets in Multiple Myeloma. BCMA, B cell maturation antigen; GPRC5D, G protein coupled receptor class C group 5 member D; SLAMF7, signaling lymphocytic activation molecule family member 7; CD, cluster of differentiation; FcRH5, Fc receptor homolog 5; TACI, transmembrane activator and CAML interactor; APRIL, A proliferation inducing ligand.

Myeloma has well characterized genomic instability, intratumoral heterogeneity, and immune-evasive properties (56–59). Thus, while it is aspirational to hope that anti-BCMA CAR T cells, perhaps with next-generation constructs, will produce long-lasting remissions for patients, pursuing other targets beyond BCMA is likely necessary to overcome complex resistance mechanisms (60). What is more, a rare but noteworthy toxicity of anti-BCMA CAR T cell therapy is treatment-associated parkinsonism, which is poorly understood, but has generated a measure of justifiable apprehension (61, 62). In this review, we aim to summarize several emerging CAR T cell therapies with novel non-BCMA targets in myeloma, focusing on targets with more extensive preclinical rationale and clinical trial data (see **Figure 1**). Notably, the scope of this review does not include other novel anti-BCMA CAR T cells in earlier stages of investigation, nor advancements in other immunotherapeutic modalities such as bispecific TCE (63–66).

## GPRC5D

G protein-coupled receptor class C group 5 member D (GPRC5D) was initially identified as a transcribed mRNA in malignant plasma cells over a decade ago, but it was not until 2019 that GPRC5D was shown to be expressed on the cell surface (67, 68). Human GPRC5D expression otherwise appeared to be limited to hair follicles and skin, which made it a potentially promising immunotherapeutic target. In their seminal paper, Smith et al. developed and characterized multiple anti-GPRC5D CAR T cell constructs with *in vitro* and *in vivo* activity and no significant cross-reactivity to other tissues (including hair and skin) in mice and non-human primates (68). Consequently, these and other anti-GPRC5D CAR T cells were rapidly translated into early phase clinical trials. The first phase 1 study reported in 2022 by Mailankody et al. described 17 heavily pre-treated patients infused with MCARH109, which contains a humanized anti-GPRC5D scFv target-binding domain and a 4-1BB co-stimulatory domain (69). CAR T cell doses ranged from 25×10^6^ to 450×10^6^ cells. Many of the toxicities were akin to prior experience with other FDA approved CAR T cell therapies including CRS (overall, 88%; grade ≥3, 6%), ICANS (overall, 6%; grade ≥3, 6%), infections (overall, 18%; grade ≥3, 12%), and cytopenias (grade ≥3, 94%). Of note, nail loss was common (65%), although this reversed without intervention in most. Rash was uncommon but reported (grade 1, 18%), as was dysgeusia (grade 1, 12%). An important discovery was the evolution of a persistent cerebellar syndrome in two patients (12%) who both received the highest dose level (450×10^6^ cells), characterized by visual fixation problems, appendicular and truncal ataxia, gait abnormalities and dysarthria. Neural imaging in both patients did not reveal any focal lesions. However, the authors noted that analysis of the Allen Brain Atlas revealed there is focal expression of GPRC5D in the inferior olivary nucleus in the human brainstem, which they hypothesized may account for this unique toxicity, although further research is required (70). In terms of efficacy, MCARH109 had an ORR of 71%, with a 35% CR rate, MRD-negative rate of 47%, and a median duration of response (DOR) of 7.8 months.

Several other groups have now reported phase 1 trial results with other anti-GPRC5D CAR T cell products (see **Table 1** for direct comparisons among reported non-BCMA targeted CAR T cell trials). Bal et al. presented results of 70 patients treated with BMS-986393 (CC-95266), which reportedly has a similar construct to MCARH109: ORR was 86%, with 38% CR; toxicities were similar to MCARH109 including two patients with a cerebellar syndrome (71). In China, Xia et al. reported results of 33 patients treated with an anti-GPRC5D CAR T cell construct with a humanized single scFv target-binding domain and a 4-1BB co-stimulatory domain (72). Patients in the study were randomized to receive all-trans retinoic acid (ATRA) in the peri-CAR T cell infusion period based on a preclinical study that posited ATRA may affect GPRC5D expression (79). In the entire cohort, the ORR was 91% with a 64% CR rate, although there was no significant difference in response noted in the ATRA exposed patients. Toxicities were comparable to prior experience with anti-GPRC5D CAR T cells, including CRS (overall, 76%; grade ≥3, 0%), ICANS (overall, 6%; grade ≥3, 3%), nail changes (27%) and skin toxicity, including palm/sole desquamation (3%). Notably, no cerebellar syndrome was reported. One patient died of cerebral hemorrhage in the setting of severe thrombocytopenia. Another group from China reported the results of ten patients treated with OriCAR-017, which is a dual epitope camelid heavy-chain only anti-GPRC5D construct (73). In the study, the ORR was 100% with a 60% CR rate. Again, toxicities were comparable to prior experience with anti-GPRC5D CAR T cells, although here with no reported ICANS or cerebellar syndrome. Finally, Li et al. reported on seven patients infused with an anti-GPRC5D CAR T cell product, with an ORR of 86%, with 43% CR, and similar toxicities to prior reports (74). Several other anti-GPRC5D CAR T cell therapy trials are registered (see **Table 2** for a summary of registered non-BCMA CAR T cell therapy trials without published results).

**Table 1:** Results from Clinical Trials with Non-BCMA CAR T cell Therapies.

Target	Product name	N	High-risk cytogenetics*†	Extra- medullary disease*	Prior lines of therapy	Prior anti-BCMA*	CRS (grade ≥3)*	ICANS (grade ≥3)*	ORR (CR)*	Sponsor	Country	Ref.
GPRC5D	MCARH109	17	76	47	6	59	88 (6)	6 (6)	71 (35)	Memorial Sloan Kettering Cancer Center	USA	(69)
GPRC5D	BMS-986393	70	46	43	–	46	84 (4)	11 (3)	86 (38)	Bristol Myers Squibb	USA	(71)
GPRC5D	–	33	39	33	4	27	76 (0)	6 (3)	91 (64)	Xuzhou Medical University	China	(72)
GPRC5D	OriCAR-017	10	60	40	6	50	90 (0)	0	100 (60)	Zhejiang University	China	(73)
GPRC5D	–	7	–	–	–	43	86 (0)	0	86 (43)	920th Hospital of Joint Logistics Support Force	China	(74)
SLAMF7/BCMA	–	16	–	38	4	13	38 (6)	0	81 (38)	Union Hospital, Tongji Medical College	China	(75)
CD38/ BCMA	–	23	–	39	4	0	87 (17)	0	87 (52)	Union Hospital, Tongji Medical College	China	(76)
CD38/ BCMA	–	16	69	50	3	0	75 (31)	–	88 (81)	Jingzhou Central Hospital	China	(77)
CD38/ BCMA	–	22	86	14	–	0	73 (27)	14 (0)	91 (55)	Tianjin First Central Hospital	China	(78)

*Reported as percentage of total patients in clinical trial.

†High risk cytogenetics defined as del(17p), t(4;14), t(14;16) and/or 1q amplification.

**Table 2:** Single-Target Non-BCMA CAR T cell Therapy Clinical Trials.

Target	ClinicalTrials.gov ID	Sponsor	Country
GPRC5D	NCT04674813	Bristol Myers Squibb	USA
GPRC5D	NCT05739188	920th Hospital of Joint Logistics Support Force of PLA of China	China
GPRC5D	NCT05219721	Tongji Hospital	China
GPRC5D	NCT05759793	Nanjing IASO Biotechnology Company	China
GPRC5D	NCT05749133	Institute of Hematology & Blood Diseases Hospital	China
GPRC5D	NCT05838131	Shanghai Changzheng Hospital	China
SLAMF7	NCT03958656	National Cancer Institute	USA
SLAMF7	NCT03710421	City of Hope Medical Center	USA
SLAMF7	NCT04499339	Wuerzburg University Hospital	Europe
SLAMF7	NCT04541368	Zhejiang University	China
CD38	NCT05442580	University of Pennsylvania	USA
CD38	NCT03464916	Sorrento Therapeutics	USA
CD138	NCT03672318	University of North Carolina – Chapel Hill	USA
BCMA/TACI (APRIL)	NCT04657861	Zhejiang University	China
BCMA/TACI (TriPRIL)	NCT05020444	Massachusetts General Hospital	USA
CD70	NCT04662294	Zhejiang University	China

Mechanisms of resistance to anti-GPRC5D CAR T cell therapy are beginning to be dissected. Unlike relapse after anti-BCMA CAR T cells, where complete BCMA loss is rare, four of six patients who had an initial response then relapsed in the study by Mailankody et al. demonstrated complete loss of GPRC5D expression (69). In one of these patients, biallelic deletions encompassing the *GPRC5D* loci was uncovered (80). Two recent publications characterized relapses after anti-GPRC5D bispecific TCE (40, 81). Both highlighted complex subclonal *GPRC5D* deletions and mutations that precipitated loss of GPRC5D expression in several patients. Fascinatingly, Derrien et al. also described a patient with acquired loss of chromatin accessibility in the promoter of *GPRC5D*, as well as more distant enhancer regions, indicating epigenetic silencing (81). Although it is not known if these mechanisms are involved in relapse after anti-GPRC5D CAR T cell therapy, these studies highlight the complex nature of myeloma tumor biology and requirement of layered treatment strategies to thwart resistance.

One obvious solution would be to concurrently or sequentially target BCMA, which appears to have heterogeneity of expression independent from GPRC5D (68). In fact, Smith et al. showed in a myeloma xenograft model of relapse mediated by BCMA loss, anti-GPRC5D CAR T cells effectively eradicated residual tumor (68). Pointing toward future approaches, a preclinical study showed that a bicistronic construct (distinct anti-BCMA and anti-GPRC5D CARs transduced via a single vector) was an optimal method of dual-antigen targeting (82). Several dual-antigen targeting concepts are being clinically tested (see **Table 3** for a summary of registered combination CAR T cell therapy trials). One study is testing pooled anti-GPRC5D and anti-BCMA CAR T cells, i.e. with MCARH109 and MCARH125, respectively (NCT05431608). There are also several trials with dual-targeted anti-GPRC5D and anti-BCMA constructs (United States: NCT06153251; China: NCT05509530, NCT05998928, NCT05325801). Trials are also underway with other anti-GPRC5D CAR T cell therapeutic combinations, including with an anti-BCMA bispecific TCE, as well as with cereblon E3 ligase modulatory drugs (NCT06121843). Preclinical approaches to improve the efficacy of anti-GPRC5D CAR T cell therapy are under investigation, such as with lysophosphatidic acid receptor modulation (83). There are also other novel approaches including anti-GPRC5D allogeneic CAR T cells and CAR natural killer (NK) cells in the pipeline (84, 85). Taken together, the encouraging safety and efficacy data from early phase anti-GPRC5D CAR T trials have propelled GPRC5D forward as the next promising therapeutic target moving closer to regulatory approval in myeloma.

**Table 3:** Combination CAR T cell Therapy Clinical Trials.

Target(s)	Co-targeting strategy	Consolidation Therapy	ClinicalTrials.gov ID	Sponsor	Country
BCMA	–	Lenalidomide	NCT03070327	Memorial Sloan Kettering Cancer Center	USA
BCMA	–	γ-secretase inhibitor (JSMD194)	NCT03502577	Fred Hutchinson Cancer Center	USA
BCMA	–	Belantamab Mafodotin	NCT05117008	Medical College of Wisconsin	USA
BCMA	–	Cevostamab (anti-FcRH5 TCE)	NCT05801939	University of Pennsylvania	USA
BCMA/CD38	Dual-targeted construct		NCT03767751	Chinese PLA General Hospital	China
GPRC5D	–	Alnuctamab (anti-BCMA TCE), mezigdomide, or iberdomide	NCT06121843	Juno Therapeutics	USA
GPRC5D/BCMA	Pooled CAR T cells	–	NCT05431608	Memorial Sloan Kettering Cancer Center	USA
GPRC5D/BCMA	Dual-targeted construct	–	NCT06153251	Juno Therapeutics	USA
GPRC5D/BCMA	Dual-targeted construct	–	NCT05998928	Wuhan Union Hospital	China
GPRC5D/BCMA	Dual-targeted construct	–	NCT05325801	Zhejiang University	China
GPRC5D/BCMA	Dual-targeted construct	–	NCT05509530	Xuzhou Medical University	China
GPRC5D/BCMA	Dual-targeted construct	–	NCT06068400	Guangzhou Bio-gene Technology Company	China
SLAMF7/BCMA	Dual-targeted construct	–	NCT05950113	UCLA Jonsson Comprehensive Cancer Center	USA

## SLAMF7

The glycoprotein cell surface receptor signaling lymphocytic activation molecule family member 7 (SLAMF7, also called CS1 or CD319) is highly expressed on malignant plasma cells. SLAMF7 is functionally important for plasma cell survival, and its discovery prompted development of the anti-SLAMF7 antibody elotuzumab, which is FDA-approved for administration in combination with lenalidomide and dexamethasone for the treatment of R/R myeloma (86–88).

Several groups developed anti-SLAMF7 CAR T cells and tested them in preclinical models (89–92). Gogishvili et al. developed an anti-SLAMF7 CAR T cell construct using the target-binding domain from elotuzumab paired with a CD28 co-stimulatory domain (90). These anti-SLAMF7 CAR T cells effectively killed myeloma cells in patient samples and a murine xenograft model. Of note, unlike BCMA or GPRC5D, SLAMF7 is expressed on other immune cell types including T cells, B cells, NK cells, monocytes, and dendritic cells. As such, the capacity for fratricide both of anti-SLAMF7 CAR T cells and other immune cells was investigated. Unsurprisingly, at the end of manufacturing, the anti-SLAMF7 CAR T cells were SLAMF7-negative/low, likely due to fratricide of SLAMF7-high cells. In co-culture with autologous immune cells, anti-SLAMF7 CAR T cells also induced fratricide of SLAMF7-high unmodified T cells, but spared SLAMF7-negative/low T cells, and only caused partial B cell depletion. Importantly, SLAMF7 negative/low unmodified T cells retained the ability to respond to viral antigens in their study.

Another group generated anti-SLAMF7 CAR T cells with a slightly different target-binding epitope (distal V2 domain), and a third-generation combination CD28 and 4-1BB co-stimulatory domain, which effectively killed malignant plasma cells (92). Interestingly, these CAR T cells were predominantly CD4+, suggesting that CD8+ CAR T cells fell victim to fratricide during *ex vivo* production. As such, they used CRISPR/Cas9 technology to delete *SLAMF7* and create fratricide resistant anti-SLAMF7 CAR T cells. Despite yielding a more balanced CD4:CD8 T cell profile, SLAMF7-deficient CAR T cells were not significantly more effective in their murine xenograft models. Taking these studies together, while the consequences of fratricide of both anti-SLAMF7 CAR T cells and other immune cells require further investigation, it appears that anti-SLAMF7 CAR T cells could be a promising therapeutic strategy for the treatment of myeloma.

Clinical trials are now assessing anti-SLAMF7 CAR T cell therapy in patients. Based on a preclinical study with a dual-targeted single-stalk CAR with anti-BCMA and anti-SLAMF7 domains, a phase 1/2 trial was initiated and the results with 16 infused patients were recently reported (75, 93). Toxicities included CRS (overall, 38%; grade ≥3, 6%), with no reported ICANS, but significant cytopenias (overall, 100%; grade ≥3, 100%), and infections (overall, 38%; grade ≥3, 31%). In terms of efficacy, the ORR was 81%, with 38% CR, and 1-year DOR was 56%. Other groups have developed different CAR constructs co-targeting BCMA and SLAMF7, and one is being clinically tested (NCT0595011) (94, 95). An allogeneic fratricide resistant anti-SLAMF7 CAR T showed promising preclinical results, but the phase 1 trial (NCT04142619) was terminated, in part due to a fatal cardiac arrest event necessitating a FDA hold, and no results are published (96). Several trials are registered with CAR T cells targeting SLAMF7 alone (United States: NCT03958656, NCT03710421; Europe: NCT04499339; China: NCT04541368).

## CD38

The discovery that the ectoenzyme CD38 was highly expressed on the cell surface plasma cells led to one of the most significant therapeutic advancements in myeloma of the past decade – the advent of anti-CD38 targeting antibodies (97, 98). The first FDA approved anti-CD38 antibody daratumumab transformed the treatment of relapsed disease and more recently has moved into the front-line treatment setting (99, 100). Besides plasma cells, CD38 is expressed on hematopoietic progenitor cells, as well as T cell subsets, NK cells, myeloid-derived suppressor cells, and regulatory B cells. Unsurprisingly, exposure to daratumumab has been shown to perturb some of these cell populations (101, 102). Nevertheless, the lack of significant organ toxicity, including paucity of cytopenias, after anti-CD38 antibody exposure underpins its value as a therapeutic target, with elevated risk of infections being the primary adverse effect associated with treatment.

A number of anti-CD38 CAR T cells have been generated and evaluated in preclinical studies (103–107). Several groups noted that expanded anti-CD38 CAR T cells were CD38-negative, likely secondary to fratricide of CD38-positive cells (103, 105). Even so, these CAR T cells effectively killed myeloma cells in model systems, in line with prior knockout mouse studies suggesting that CD38 is generally dispensable for T cell function (108). Interestingly, anti-CD38 CAR T cells generated by Glisovic-Aplenc et al. did not undergo fratricide, likely due to a protective effect mediated by the CAR construct itself (107). Importantly, although anti-CD38 CAR T cells reduce CD34+ CD38+ hematopoietic progenitor cells *in vitro* and *in vivo*, several groups noted anti-CD38 CAR T cells do not have a significant effect on downstream hematopoietic lineages, suggesting the CD34+ CD38-low/negative compartment is sufficient to recapitulate hematopoiesis (105, 107). Obviously, as these therapies move into human testing, close observations of the effect of anti-CD38 CAR T cell exposure on the number and function of immunologic and hematopoietic cells in patients is required.

Several Chinese groups have now reported results of phase 1 trials with anti-CD38 CAR T cell therapy, albeit in combination with anti-BCMA therapy (76–78). Mei et al. designed a dual-targeted single-stalk CAR with anti-CD38 and anti-BCMA domains and a 4-1BB co-stimulatory domain (76). Because of concerns that CD38 targeting would cause significant hematopoietic toxicity, they selected a scFv with reduced binding affinity to CD38 relative to the anti-BCMA domain. These CAR T cells were infused in 23 patients and outcomes were reported. Most common toxicities included CRS (overall, 87%; grade ≥3, 17%), with no reported ICANS, although many patients had significant cytopenias (overall, 96%; grade ≥3, 87%), which persisted longer than a month in a considerable proportion. Two patients died: one from infection, one from cerebral hemorrhage. The reported ORR was 87%, with 52% CR, and 1-year DOR of 76%. Another group performed a trial with a similar dual-targeted construct (77). In 16 infused patients, toxicities were similar to the prior report with CRS and cytopenias; notably, one patient who had a CR died of infection in the setting of prolonged CRS and persistent cytopenias secondary to hemophagocytic lymphohistiocytosis. The reported ORR was 88%, with 81% CR, and several of the responses lasted over a year, although DOR was not formally reported. Finally, a third group reported the results with a pooled infusion of separate anti-CD38 and anti-BCMA CAR T cells (78). In 22 infused patients, toxicities were consistent with prior reports, although notably two patients died of refractory CRS. The ORR was 91%, with 55% CR. Another trial in China concurrently targeting CD38 and BCMA is registered (NCT03767751).

Taken together, the major caveat in interpreting these trials is that the observed toxicity and responses attributable to the anti-CD38 component of therapy is unclear, given all were combined with anti-BCMA constructs. Moreover, many patients were early in their treatment course, all were naïve to anti-BCMA therapeutics, many had no prior daratumumab exposure, and a subset were even naïve to standard up-front therapies including immunomodulatory drugs, making comparison to other trials dubious. Early phase clinical trials targeting anti-CD38 alone in the appropriate clinical context with correlative studies are necessary. In fact, several trials are currently registered in the United States with anti-CD38 CAR T cells (NCT03464916, NCT05442580). There are other novel approaches in the pre-clinical pipeline targeting CD38 as well, including anti-CD38 CAR NK cells (109–111).

## CD138

The transmembrane proteoglycan CD138 (also called Syndecan-1) is expressed on terminally differentiated B cells and plays a key role in plasma cell survival (112). A priori, its utility as a therapeutic target is theoretically limited by CD138 expression on other cell types including subsets of epithelial and endothelial cells (113). With these potential pitfalls in mind, anti-CD138 CAR T cells have been preclinically developed and characterized by several groups (114, 115). Notably, the anti-CD138 CAR T cells described by Sun et al. did not lyse endothelial or epithelial cell lines in co-culture experiments (114). A clinical trial with five patients treated with anti-CD138 CAR T cells conducted in China was reported in 2016 (115). Although no objective responses were observed, there was no standard reporting of toxicities, limiting the interpretation of this study. Another clinical trial with anti-CD138 CAR T cells is enrolling in the United States (NCT03672318). Preclinical studies to optimize anti-CD138 CAR T cell therapy are also underway. For example, a recent publication detailed the design and optimization of a dual-split CAR construct with anti-CD38 and anti-CD138 domains, whereby CAR T cell activation occurred only in the presence of both antigens (116). These dual-targeted CAR T cells were effective at eliminating malignant plasma cells, but importantly spared other cell types including hematopoietic precursors.

## FcRH5

Fc receptor-homolog 5 (FcRH5) is a surface antigen that is highly expressed on plasma cells and has emerged as an exciting target for immunotherapy in myeloma (117). FcRH5 expression in human tissues otherwise seems to be limited to select B cell subsets. Interestingly, the corresponding gene *FCRL5* is located on chromosome 1q, thus FcRH5 is highly expressed in patients with amplification of 1q21, a poor-risk marker in myeloma (118, 119). Cevostamab is an anti-FcRH5 bispecific TCE currently in early phase clinical trials that has shown promising responses with limited toxicity in patients (120). Preclinically, an anti-FcRH5 CAR T cell was recently developed and shown to eliminate myeloma cells *in vitro* and *in vivo*, including in a model of BCMA antigen loss (119). The authors also developed a dual-targeted single-stalk anti-BCMA and anti-FcRH5 CAR T construct that appeared promising. Although no anti-FcRH5 CAR T cell clinical trials are registered presently, it seems likely to be pursued.

## CD229

The SLAM family receptor CD229 (also called Ly-9), like SLAMF7, is highly expressed on plasma cells (121). Anti-CD229 CAR T cells were recently described and shown to be highly effective at eradicating myeloma cells in preclinical models (122). Importantly, CD229 expression is otherwise limited to T cells and to a lesser extent B cells, but, unlike SLAMF7, is not appreciably expressed on NK cells, monocytes, or dendritic cells. Thus, similar to the aforementioned reports with anti-SLAMF7 CAR T cells, fratricide of unmodified lymphocytes emerged as a potential downside of anti-CD229 CAR T cell exposure (90, 122). To address this potential issue, Vander Mause et al. recently published a novel approach whereby the affinity of the anti-CD229 target-binding domain was slightly reduced, which, in conjunction with modifying the CAR construct to over-express the transcription factor c-Jun, generated CAR T cells with a favorable immunophenotype that targeted myeloma cells but spared healthy unmodified lymphocytes (123). To date, there are no clinical trials registered with anti-CD229 CAR T cells.

## APRIL

A Proliferation-Inducing Ligand (APRIL) is an endogenous ligand of BCMA. APRIL also binds to Transmembrane Activator and CAML Interactor (TACI), another tumor necrosis factor superfamily receptor that, similar to BCMA, is expressed almost exclusively on plasma cells (124, 125). To take advantage of its ability to bind both BCMA and TACI with high affinity, CAR T cells with a target-binding domain derived from APRIL itself were developed by several groups (125, 126). APRIL-based CAR T cells effectively killed myeloma cells in preclinical experiments, including models of BCMA antigen loss (125, 126). However, in a phase 1 clinical trial with monomeric APRIL-based CAR T cells, the response rate was 46%, which was disappointing compared to other contemporaneous anti-BCMA CAR T cell trials (127). Subsequent studies revealed that APRIL-based CAR T cells exhibited inadequate T cell activation, secreted lower than expected levels of cytokines and thus had poor *in vivo* expansion, perhaps explaining the tepid results. Schmidts et al. devised trimeric APRIL-based CAR T cells with the goal of more closely approximating the natural APRIL ligand conformation (125). These “TriPRIL” CAR T cells had enhanced functionality compared to monomeric APRIL-based CAR T cells and are being clinically tested (NCT05020444). The same group also recently designed a more traditional scFv-based dual-targeted anti-TACI and anti-BCMA CAR T construct that was highly effective in preclinical models, including when expression of either antigen was lost (128).

## Other targets

Clinical trials with non-BCMA targeted immune effector cell therapies that have not shown remarkable efficacy to date include the following:

- κ light chain: Taking advantage of the κ light chain-restricted nature of a considerable proportion of B cell tumors, a phase 1 trial with anti-κ light chain CAR T cells was published, although there were no responses demonstrated in myeloma patients (129).
- NKG2D: Given a wide range of tumors including myeloma cells upregulate NKG2D ligands on the cell surface, several trials with anti-NKG2D ligand CAR T cells were reported, although no responses were observed (130, 131).
- CD19: The use of anti-CD19 CAR T cell therapies given immediately after autologous hematopoietic cell transplantation (AHCT) has been explored, with the idea that CD19 may be expressed on a tumor propagating myeloma stem cell compartment that could be putatively eradicated (132–134). Results with this approach were mixed, and it is difficult to isolate the effect of the anti-CD19 CAR T cells *per se*.
- NY-ESO-1: A trial with anti-NY-ESO-1 TCR-engineered T cells given after autologous HCT was reported, with similar caveats to anti-CD19 CAR T cell therapy trials, although correlative studies suggested potential biologic activity associated with response (135, 136).

Furthermore, a number of preclinical studies have defined other CAR T cell targets in myeloma, including integrin β_7_, CD44 splice isoform variant 6, CD56, CD70, Lewis Y antigen, and leukocyte immunoglobulin-like receptor subfamily B member 4 (137–142).

## Discussion

The early experience with ide-cel and cilta-cel underpinned the potency of anti-BCMA CAR T cell therapy in multiple myeloma, and triggered a rapid evolution in the treatment of the disease (15, 19). In April 2024, the FDA granted regulatory approval for use of ide-cel and cilta-cel in the early relapsed setting (1-2 lines of prior therapy), and consequently patients are increasingly being treated with anti-BCMA CAR T cell therapy earlier in their disease course. Furthermore, upcoming randomized phase 3 clinical trials are evaluating anti-BCMA CAR T cells as a component of front-line therapy for newly diagnosed myeloma. For example, cilta-cel is being tested as consolidation in transplant-ineligible patients (CARTITUDE-5, NCT04923893). Cilta-cel is also being compared with AHCT as consolidation after induction for transplant-eligible patients (CARTITUDE-6, NCT05257083). In addition, ide-cel is being tested as a consolidation therapy for patients with a sub-optimal response after AHCT (KarMMa-9, NCT06045806). While depth and duration of response are key endpoints for these clinical trials, a focus on treatment-related toxicities including long-term neurologic toxicities as well as secondary malignancies should be prioritized.

The ideal sequencing of anti-BCMA CAR T cell therapy with other novel immunotherapies is not known. For example, in patients exposed to anti-BCMA bispecific TCE, Lee et al. described acquired mutations in the *BCMA* extracellular domain that abrogated drug binding (40). However, to date there are no validated assays to test for these mutations in the clinic. At minimum, BCMA expression should be tested by immunohistochemistry prior to anti-BCMA CAR T cell therapy in patients exposed to prior BCMA-directed treatments. Overall, the challenge of relapse after treatment with anti-BCMA therapies is becoming increasingly relevant and urgently requires innovative approaches.

Novel non-BCMA targeted CAR T cell therapies are a potential solution to combat baseline intratumoral antigen heterogeneity, acquired antigen loss and immune exhaustion (8, 60, 143, 144). For example, CAR T cells targeting GPRC5D are furthest in the developmental pipeline, and to date have shown promising safety and efficacy, including in patients who relapsed after prior anti-BCMA therapies (69, 71). The optimal sequencing of anti-BCMA and anti-GPRC5D directed treatments is not known. Early data suggests that surface expression as well as acquired mutations in BCMA and GPRC5D appear to be the consequence of selective pressures from specific therapies and therefore largely independent of each other (40). Unlike *BCMA*, it appears *GPRC5D* may be genetically lost more frequently, so testing for expression by immunohistochemistry prior to anti-GPRC5D CAR T cell therapy is prudent, especially if patients have prior exposure to anti-GPRC5D bispecific TCE (40, 69, 80, 81). A number of other promising targets of interest were highlighted in the review and in the early stages of clinical evaluation.

There are many pressing questions as CAR T cell therapies in myeloma are refined. Understanding the co-expression patterns of multiple antigens such as BCMA, GPRC5D and FcRH5 will be relevant as combination therapies, e.g., dual-targeted CAR T cells, are tested in the clinic (145). Given that antigen density on tumor cells is a crucial factor mediating CAR T cell resistance, the effect of tumor mutations such as *KRAS* and *TP53* on surface antigen expression could be informative (37, 146, 147). Moreover, as a potential combination therapy with anti-BCMA CAR T cells, it is noteworthy that γ-secretase inhibition may affect expression of other antigens as well (23, 148). Several recent presentations at the American Society of Hematology 2023 meeting highlighted ongoing work to decipher the immunologic landscape of response and relapse after anti-BCMA CAR T cells (51–54). These analyses build on the growing understanding of optimal CAR T cell immune characteristics and will be required for CAR T cell therapies targeting other antigens. Further, as trials investigate using bispecific TCE to amplify responses after CAR T cell therapy, studies deciphering how TCE impact the immunologic milieu are necessary (149, 150). Much work remains to fine-tune the CAR T cell approach in cancer more broadly as well (8, 151, 152). Perhaps the advent of combinatorial logic-gated CAR T cells will further open the search for other relevant targets (152, 153). Although not discussed in this review, alternative immune effector cell therapies such as CAR NK cells or CAR macrophages may have certain advantageous properties and could make an impact as well (154–157).

In summary, the future of CAR T cell therapy in myeloma is bright as constructs aimed at targets beyond BCMA enter the treatment landscape. Rigorously designed CAR T cell clinical trials with accompanying correlative studies are necessary to generate hypotheses that can be investigated in translational laboratories, particularly to elucidate mechanisms of relapse and optimal sequencing of therapies aimed at different targets. Moreover, ongoing work to interrogate the safety profile of these CAR T cell therapies is vital, as both anti-BCMA and anti-GPRC5D CAR T cells have raised concerns about rare but serious long-term neurologic toxicities which are poorly understood (61, 69). Future practical considerations of rechallenging with CAR T cell therapy, albeit against different antigens, include the unknown cumulative clinical consequences of repeated exposure to cytotoxic lymphodepleting chemotherapy, as well as potential diminishing immunologic fitness after multiple lines of prior therapy affecting the manufacturing quality of CAR T cell products. Finally, the financial toxicities associated with sequential administration of highly costly cellular therapeutics is uncharted outside of clinical trial settings and will likely be a major challenge. With all these caveats in mind, the rapid pace of innovation suggests highly active CAR T cell therapies leading to more durable remissions for patients with myeloma could be on the horizon.

## Author contributions

KM: Writing – original draft, Writing – review & editing. HH: Writing – original draft, Writing – review & editing. SR: Writing – original draft, Writing – review & editing.
